# The fate of patients with intermittent claudication in the 21st century revisited – results from the CAVASIC Study

**DOI:** 10.1038/srep45833

**Published:** 2017-04-03

**Authors:** Barbara Rantner, Barbara Kollerits, Johannes Pohlhammer, Marietta Stadler, Claudia Lamina, Slobodan Peric, Peter Klein-Weigel, Hannes Mühlthaler, Gustav Fraedrich, Florian Kronenberg

**Affiliations:** 1 Division of Genetic Epidemiology, Department of Medical Genetics, Molecular and Clinical Pharmacology, Medical University of Innsbruck, Innsbruck, Austria; 2 Department of Vascular Surgery, Medical University of Innsbruck, Innsbruck, Austria; 3 3rd Medical Department of Metabolic Diseases and Nephrology, Hietzing Hospital, Vienna, Austria; 4 King’s College London, Diabetes Research Group, London, United Kingdom; 5 Clinic for Angiology, HELIOS Klinikum Berlin-Buch, Berlin, Germany; 6 Clinic for Surgery, Bezirkskrankenhaus Schwaz, Schwaz, Austria

## Abstract

Patients with intermittent claudication carry a high risk for cardiovascular complications. The TransAtlantic Inter-Society Consensus (TASC) Group estimated a five-year overall mortality of 30% for these patients, the majority dying from cardiovascular causes. We investigated whether this evaluation is still applicable in nowadays patients. We therefore prospectively followed 255 male patients with intermittent claudication from the CAVASIC Study during 7 years for overall mortality, vascular morbidity and mortality and local PAD outcomes. Overall mortality reached 16.1% (n = 41). Most patients died from cancer (n = 20). Half of patients (n = 22; 8.6%) died within the first five years. Incident cardiovascular events were observed among 70 patients (27.5%), 54 (21.2%) during the first five years. Vascular mortality was low with 5.1% (n = 13) for the entire and 3.1% for the first five years of follow-up. Prevalent coronary artery disease did not increase the risk to die from all or vascular causes. PAD symptoms remained stable or improved in the majority of patients (67%). In summary, compared to TASC, the proportion of cardiovascular events did not markedly decrease over the last two decades. Vascular mortality, however, was low among our population. This indicates that nowadays patients more often survive cardiovascular events and a major number dies from cancer.

Peripheral arterial disease (PAD) belongs to atherosclerotic diseases and is predictive for future cardiovascular events. Since arterial disease normally affects a variety of vascular beds patients carry a significant risk for stroke, myocardial infarction and other cardiovascular events. Literature from the late 1990s revealed that survival of PAD patients is mainly influenced by cardiovascular complications^1^,^2^. Especially patients with concomitant coronary artery disease carry a high risk for life-threatening complications. In the early 1960s Begg and Richards as well as Juergens already recognized that PAD patients in the state of intermittent claudication (IC) with clinical or electrocardiographic signs of coronary ischemia had a survival probability comparable to patients who had survived a myocardial infarction^3^,^4^.

In their consensus report in the year 2000, the TransAtlantic Inter-Society Consensus (TASC) Group described the five year fate of a patient with intermittent claudication: 5–10% of these patients were expected to suffer from non-fatal cardiovascular events, 30% were supposed to die mostly due to vascular reasons and only 55–60% of patients were expected to be alive without new cardiovascular complications after 5 years of observation^5^. The updated version of the TASC consensus document from 2007 - TASC II - contained slightly changed complication estimates predicting about 20% non-fatal cardiovascular events and proportion of deaths ranging between 10% and 15%, predominantly due to vascular causes^6^. The American College of Cardiology/American Heart Association (ACC/AHA) guidelines of the year 2005 presented comparable numbers of non-fatal cardiovascular events as described in the TASC II document during a five year period. However, they predicted 15–30% events of death with vascular complications again being causative for the majority of deaths^7^.

Furthermore, all three documents presented nearly similar numbers for disease progression in the leg. TASC I assumed that 75% of patients with intermittent claudication would stabilize or improve. From the remaining 25% who deteriorate only 5% would require an intervention and 2% would need a major amputation^5^. The ACC/AHA guidelines and the updated TASC II document indicated a stabilization of disease in 75–80% of patients. Among those who deteriorate, 10–20% would still suffer from intermittent claudication, whereas 5–10% would develop critical limb ischemia. The proportion of amputation would still be as low as 1–2%^6^,^7^.

On the basis of the TASC I consensus document we initiated the CAVASIC (CArdioVAScular disease in patients with Intermittent Claudication) Study in 2002 to retrace these data in a population of well-defined PAD patients with intermittent claudication. The total prospective follow-up time added up to a median of seven years and was considered for the main outcome analysis of all primary and secondary endpoints. For better comparability with the TASC documents we separately present data for the first five years of follow-up to calculate incidence rates for overall mortality and cardio- and cerebrovascular events. The remaining years of follow-up were also considered for incidence rate analysis.

We tried to answer the following question with the study at hand: are we able to confirm the prognosis of a patient with intermittent claudication as reported in the TASC consensus statement or did the fate of these patients significantly change over the years?

## Methods

### Study participants and study design

The CAVASIC (CArdioVAScular disease in Intermittent Claudication) Study is a case-control study with a prospective follow-up investigation of patients to identify cardiovascular risk factors in male patients with intermittent claudication^8^,^9^,^10^. Recruitment of patients and controls was carried out in 2 clinical centers between 2002 and 2006: the Department of Vascular Surgery, Medical University of Innsbruck, and the 3^rd^ Medical Department of Metabolic Diseases and Nephrology, Hietzing Hospital, Vienna, Austria.

Inclusion criterion for patients (n = 256) was a history of intermittent claudication (PAD IIa or IIb according to the criteria of Fontaine) irrespective of any former treatment procedure (bypass surgery or intervention). Patients who presented with acute or critical limb ischemia (Fontaine III or IV), impaired kidney function with serum creatinine >1.5 mg/dL, malignancy, previous organ transplantation, and therapy with nicotinic acid or corticosteroids were excluded from the study. Prevalent cardiovascular disease (CVD) was no exclusion criterion. Prevalent CVD was defined as documented cardiovascular events or procedures, such as myocardial infarction, percutaneous transluminal coronary angioplasty, aortocoronary bypass surgery, and/or coronary angiography with proven coronary stenosis ≥50 or stroke/TIA. At the time of enrollment none of the patients showed signs of acute illnesses or inflammatory processes.

To minimize the interobserver bias, all clinical examinations and interviews were accomplished by one physician in each of the two clinical centers. The physician was specially trained for the required methods and standardized questionnaires were used (Rose Questionnaire for angina pectoris and myocardial infarction and Edinburgh questionnaire for the assessment of symptomatic peripheral arterial disease).

From the 214 patients with symptomatic PAD still alive at the follow-up investigation^11^,^12^, interviews based on a standardized questionnaire were performed at the respective medical center (n = 181) or by telephone survey where a visit in the outpatient ward was not possible (n = 3). These examinations were done by one specially trained medical doctor and one study nurse at each participating center.

### Ethical approval and informed consent

The examination protocols of the CAVASIC Study were approved by the Ethics Committee of the Medical University of Innsbruck and by the Ethics Committee of the city of Vienna. All methods were carried out in accordance with the approved guidelines and the Declaration of Helsinki. Written informed consent was obtained from each study participant.

### Baseline and vascular examination and other phenotypic characterization

For the measurement of the ankle-brachial index (ABI) the systolic blood pressure was measured once on both arms. Two additional measurements were carried out on the arm with the higher value and these were averaged. On the lower extremity 3 measurements of the systolic blood pressure were made for each artery (dorsalis pedis artery and tibialis posterior artery, on both the left and right ankles) and the mean of the second and third for each site was calculated. For ABI calculation the mean of the lower extremity sites was divided by the averaged systolic blood pressure of the arm. The lowest ABI value from the 4 sites was used for further analysis^13^,^14^.

The Edinburgh questionnaire was applied to assess symptomatic intermittent claudication^15^. Additional examinations for IC diagnostic were pulse volume recording, treadmill examination, ultra sound scanning and magnetic resonance imaging of the arteries of the lower extremity or computed tomography and conventional angiography depending on availability and indication.

A diagnosis for type 2 diabetes mellitus (T2DM) was made if fasting plasma glucose concentrations exceeded 126 mg/dL and/or the patient was on current anti-diabetic medication. Hypertension was defined as a systolic BP ≥140 mmHg and/or a diastolic BP ≥90 mmHg and/or current treatment with antihypertensive drugs. The glomerular filtration rate (GFR) was calculated based on the CKD-EPI equation^16^.

### Laboratory measurements

The following parameters were measured routinely in serum during the patients outpatient ward visit: total, LDL and HDL cholesterol as well as triglycerides were measured by enzymatic colorimetric tests on a P800 analyzer (Modular, Roche Diagnostics), C-reactive protein (CRP) by immunoturbidimetry, glucose by an enzymatic UV test (P800 analyzer; Modular; Roche Diagnostics), serum and urine albumin by a nephelometric method (BN II; Siemens), and serum creatinine by the Jaffé method (kinetic colour assay) which was corrected for unspecific protein reactions by a factor of −26.5 μmol/L. HbA1c was measured using an immunoturbidimetric Latex-assay (Integra; Roche Diagnostics) and was standardized according to the DCCT/NGPS.

### Follow-up examination

In the CAVASIC Study PAD patients with intermittent claudication (n = 256) were prospectively followed to collect data on overall mortality and clinical endpoints (fatal and non-fatal cardiovascular events as well as local PAD outcome). Finally, follow-up information was available in 255 patients (median follow-up time 7 years, interquartile range (5 to 8 years)), and 41 patients died. Face-to face interviews including follow-up examinations were performed in 184 of 214 patients (86%) and hospital charts were available in all 255 patients (100%). Data concerning local PAD outcome were available in 254 patients. One patient was excluded from the local PAD outcome analyses due to missing of this specific information in hospital charts.

### Clinical endpoints

Overall mortality (primary endpoint), incident fatal and non-fatal cardiovascular events as well as local PAD outcome (both secondary endpoints) were considered as clinical endpoints, respectively. Autopsy reports, medical reports, death certificates or information reported by general practitioners were additionally used to verify the date of death and/or the causes of death as well as of all further reported clinical events. Cardiovascular mortality was specified according to the ICD10 Codes I00-I99, death due to cancer based on ICD10 C00- D48 and infectious mortality by codes ICD10 A00-B99.

Major cardiovascular events were defined as non-fatal myocardial infarction (MI), ischemic cerebral infarction and cardiovascular death. Non-fatal MI was diagnosed by clinical symptoms, adequate changes in electrocardiography and an increase in cardiac enzymes. In all non-fatal MI cases additionally medical records of coronary angiographies (CAGs) with or without subsequent percutaneous transluminal coronary angioplasty (PTCA) were available. Ischemic cerebral infarction was diagnosed by clinical appearance, computer tomography and/or magnetic resonance imaging.

Minor cardiovascular events comprised elective PTCA, aortocoronary bypass and angiographically proven coronary stenosis ≥50%. Transient ischemic attack (TIA) and carotid endarterectomy (CEA) were furthermore classified among minor CVD events.

For local PAD outcome patients were categorized as follows: 1) staying stable or improve and 2) deteriorate and needing intervention. Patients with disease progression reported a shorter maximum walking distance confirmed by treadmill examination or they even developed rest pain or ulcerations. They consecutively underwent a vascular intervention (endovascular treatment, operative treatment or combination of both treatment techniques). Two different local PAD endpoints were assessed: The first intervention during follow-up was documented as “*first event”*, no matter what type of intervention was carried out (endovascular treatment: percutaneous transluminal angioplasty (PTA), PTA plus stent implantation, thrombolysis; operative treatment: thrombendarterectomy of the common femoral artery and bifurcation, primary and re-do bypass surgery, bypass thrombectomies, thrombectomies of native vessels following embolic occlusion or complex procedures combining operative and endovascular treatment, i.e. thrombendarterectomy of the common femoral artery, intraoperative PTA/stent of the iliac or femoral vessels, and minor or major amputations). Additionally, major PAD events during follow-up were defined as surgical treatment for revascularization (thrombendarterectomy of the common femoral artery and bifurcation; primary and re-do bypass surgery of aorto-iliac arteries, femoro-popliteal arteries or bypass to a below the knee artery; complex procedures combining operative and endovascular treatment, as well as minor and major amputations). Not included in the major endpoint definition were thrombectomies of preexisting bypasses or native vessels following embolic occlusions.

### Statistical methods

Categorical data were compared by *χ^2^*-test, and continuous parameters by unpaired *t*-test or Wilcoxon rank-sum test in case of non-normally distributed variables. Incidence rates (per 100 person-years) were presented for overall mortality, minor and major cardiovascular complications for the first 5 years of follow-up and in addition for the entire follow-up period. For minor and major cardiovascular complications, only the first occurrence of the respective event was counted. Furthermore, 95% Wald confidence limits for incidence rates were presented.

Estimated cumulative incident curves were set up for the total patient group and the entire follow-up period on primary and secondary outcomes and additionally for those with and without an intervention at baseline for local PAD outcome (shown separately for first and major PAD event during follow-up). For all outcome variables except overall mortality, all other events that were not of interest for the respective analysis were treated as competing risks. Cox regression analysis was done to calculate hazard ratios (HRs) and corresponding 95% confidence intervals for overall mortality. For vascular mortality as well as incident cardiovascular endpoints, competing risks regression was performed to calculate cause-specific hazard ratios. All regression models were adjusted for age. The proportional hazards assumption of the Cox model was tested by χ^2^test based on Schoenfeld residuals. A two-sided p-value < 0.05 was considered statistically significant. All analyses were conducted with SPSS version 21.0 (IBM SPSS Software) and R statistical software, version 3.1.3.

## Results

### Baseline characteristics

Table 1 shows the baseline demographic and clinical data of the entire study population as well as stratified for those with and without prevalent cardiovascular disease. At baseline 72 patients had prevalent CVD and 183 were free of CVD. PAD patients with concomitant CVD were significantly older, had a higher body mass index, more often presented with hypertension and had significantly lower total, LDL and HDL cholesterol levels as well as a lower eGFR. They were more often treated with statins, beta blockers and angiotensin converting enzyme (ACE) inhibitors.

### Overall mortality, cardio- and cerebrovascular morbidity and mortality

**Figure 1:**
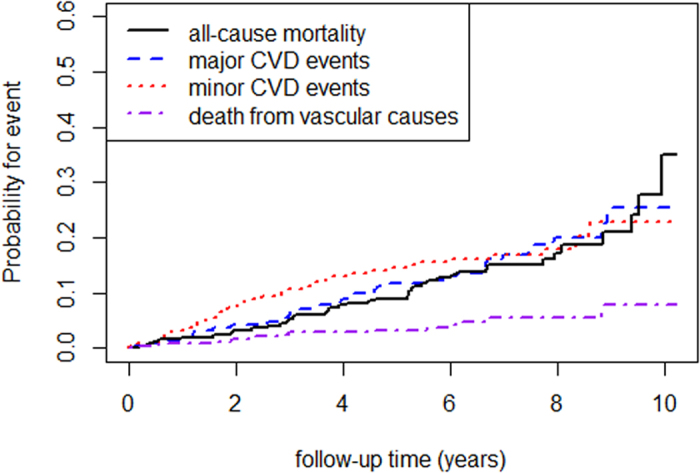
Cumulative incident curves illustrating probability for event (all-cause mortality, minor and major cardiovascular events) for the entire study population for the whole follow-up period.

Figure 1 shows the cumulative incidence (overall mortality, minor and major CVD events) for the whole study population for the entire follow-up period. Twenty-two patients (8.6%) died during the first five years of observation, and forty-one (16.1%) during the entire follow-up period (median 7 years). The majority of patients died from cancer (n = 20, 7.8%), only 13 patients due to vascular complications (5.1%). Three additional patients died from infectious disease and another five due to unknown reasons. Table 2 gives detailed information on causes of death of the entire study population for the first 5 years of follow-up and the entire follow-up period. Within the first five years we observed an overall mortality rate of 1.88 (95% CI: 1.10–2.67) per 100 person-years and 2.46 (95% CI: 1.71–3.21) for the entire follow-up period, respectively.

**Figure 2:**
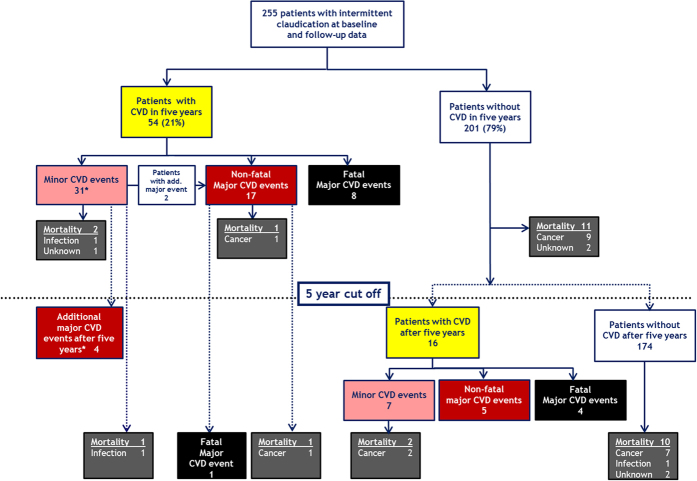
Mortality and cardiovascular complications in patients with intermittent claudication within five years (upper part of the graph) and additional events thereafter (below dotted line). *31 patients suffering from a minor cardiovascular event within the first five years consecutively had major cardiovascular events within the first five years of follow-up (n = 2), and in the remaining follow-up period (n = 4).

Figure 2 summarizes the fate of patients with intermittent claudication and provides information about causes of death and cardiovascular complications. Of the 255 patients, 54 (21%) suffered from incident cardiovascular complications during the five year period, including 25 major (17 non-fatal and 8 fatal) and 31 minor CVD events. Two patients with initial minor CVD event consecutively suffered from a major CVD event during the first five years of follow-up. Four out of 31 patients with a minor CVD event within the first five years experienced a major CVD event after the five year cut-off (Fig. 2). From the patients without CVD within the first five years (n = 201), 16 experienced incident cardiovascular events during the remaining follow-up time, 9 of them being major (5 non-fatal and 4 fatal) and 7 minor. From all patients who experienced a major fatal or non-fatal CVD event during follow-up (25 before and 9 plus 4 after the five years) 15 died, the majority (n = 13) due to vascular causes. In contrast, among all patients without CVD events, cancer mortality (n = 16 out of 21) was the predominant cause of death (Table 2).

Table 3 gives detailed information about cumulative cardiovascular events within the first five years of observation and the entire follow-up period.

Major CVD incidence rates in the first five years of follow-up were 2.54 (95% CI: 1.55–3.54) per 100 patient years and 2.76 (95% CI: 1.88–3.64) per 100 patient-years in the entire follow-up period. Minor CVD incidence rates were 3.26 (95% CI: 2.11–4.41) per 100 patient-years in the first five-years and 2.86 (95% CI: 1.95–3.77) per 100 patient-years in the entire follow-up time period, respectively.

### Overall mortality, cardio- and cerebrovascular morbidity and mortality in patients with and without prevalent cardiovascular disease

First, the age-adjusted Cox regression model on overall mortality did not violate the proportional hazards assumption: PAD patients with concomitant CVD showed a clear trend for a higher risk to die from all causes compared to those PAD patients without CVD, however, without reaching statistical significance: HR 1.66, 95%CI (0.89–3.10), p = 0.11. The same holds true for death from vascular causes: Cause-specific HR 2.67, 95%CI (0.91–7.83), p = 0.07. The risk of suffering from incident cardiovascular events was also not significantly different between the two groups: Cause-specific HR 1.43, 95%CI (0.87–2.35), p = 0.16.

### Local PAD outcome in patients with intermittent claudication

**Figure 3:**
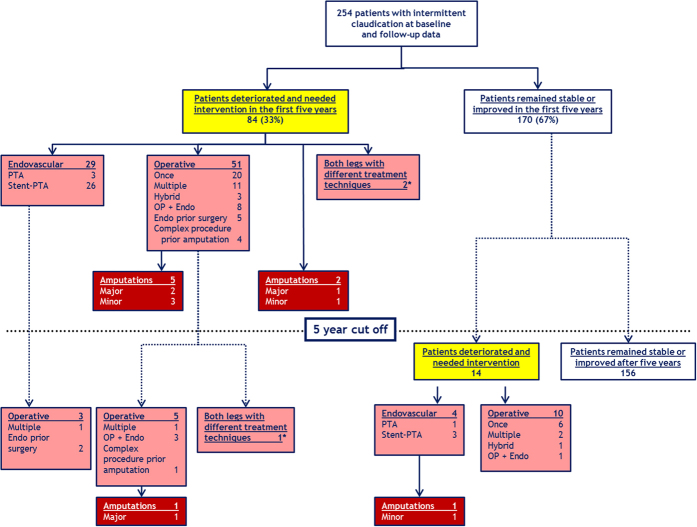
Local PAD outcome of patients with intermittent claudication within the first five years of follow-up (upper part of the graph) and additional peripheral interventions thereafter (below dotted line). *3 patients had different treatment techniques on both legs: all these patients were symptomatic on both legs when they were enrolled in the study. Two patients received endovascular treatment on one leg and underwent surgery on the other one (within the first five years of follow-up). The third patient had multiple surgeries on one leg (within the first five years of follow-up) and received endovascular treatment on the second leg (after the five year cut-off).

Follow-up information for this outcome was available in 254 patients. Figure 3 shows the course of arterial disease and local PAD outcome of 254 patients for the five year follow-up period and thereafter below the dotted line. 84 (33%) patients deteriorated and needed intervention within the five year follow-up. The majority of them (n = 51) was treated operatively, some in combination with endovascular treatment techniques. Further 29 patients were exclusively treated by endovascular methods. We observed three major and four minor amputations among these 84 patients. Another 14 patients (5.5%) deteriorated and needed peripheral vascular intervention only after the first five year follow-up period. Four of these patients underwent endovascular treatment. One of them consecutively had a minor amputation. The remaining 10 patients were treated by surgery, in most cases only once (Fig. 3). Nine patients with peripheral treatment within the five years further showed disease progression and needed additional treatment in the remaining follow-up time. In one case a major amputation was necessary. Overall, 9 patients (3.5%) needed an amputation (Fig. 3).

**Figure 4:**
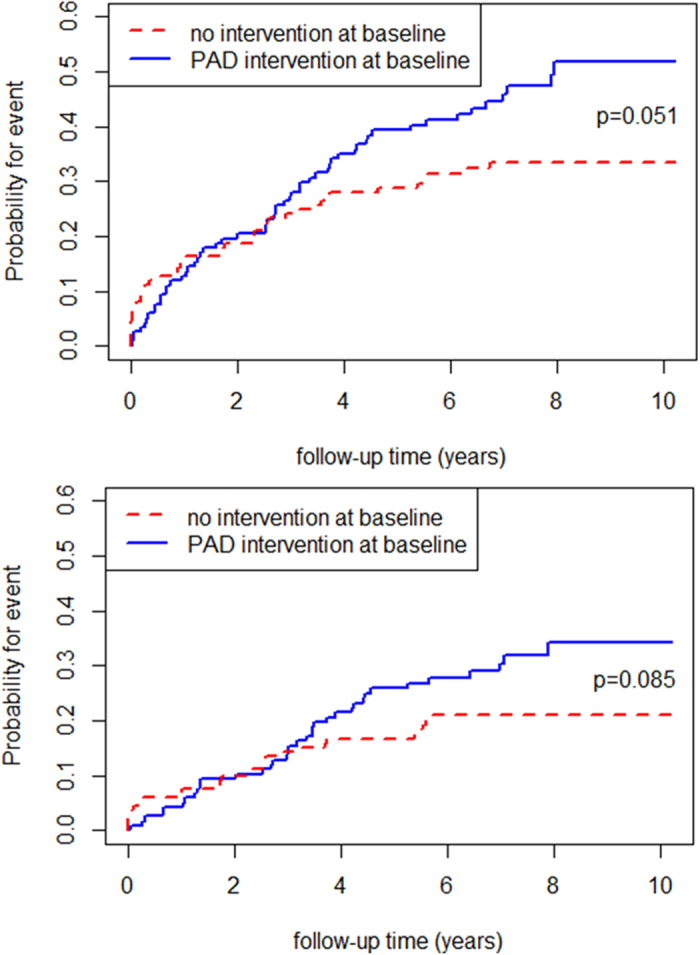
(A) Cumulative incident curves for first PAD event during follow-up comparing those with and without PAD intervention at baseline. A slightly higher risk to get a PAD intervention during follow-up for those patients with an intervention at baseline compared to those without was observed (p = 0.051). (**B**) Cumulative incident curves for major PAD events during follow-up comparing those with and without PAD intervention at baseline. The risk to get a major PAD intervention during follow-up was marginally higher for patients with an intervention at baseline compared to those without but did not reach statistical significance (p = 0.085).

In a next step we categorized patients into two groups depending on the treatment status of peripheral arteries at baseline. 118 patients had already undergone an endovascular or surgical intervention when they were enrolled in the study and 136 patients were treated conservatively at the time of the baseline investigation. There was a time-dependent effect of endovascular or surgical intervention at baseline on the risk for PAD intervention procedures during follow-up. Until the third year of follow-up, no difference in cumulative incident curves was observed, only thereafter these curves started to differentiate. This is reflected by a slightly increased risk for PAD intervention during follow-up for those with baseline endovascular or surgical interventions (Fig. 4). Overall, there is a higher risk for those patients with a PAD intervention at baseline compared to those without to get a first PAD event during follow-up (p = 0.051), which is barely not significant, though. This difference between the two groups was less pronounced when only considering major PAD events as outcome (p = 0.085). The same was reflected by age-adjusted cause-specific hazard ratios for first PAD event (HR 1.48, 95% CI (0.993–2.21), p = 0.093) and major PAD event (HR 1.54, 95% CI (0.93–2.57), p = 0.096), respectively.

## Discussion

Over a long period of time PAD patients with intermittent claudication were thought to have a limited life expectancy due to concomitant cardiovascular disease with a high number of major and minor cardiovascular complications. We aimed to elucidate this question within the CAVASIC Study and made the following observations:

1. The overall mortality rate slightly increased when the rate for the first five years was contrasted with the entire observation period (1.88 versus 2.46 per 100 person-years). For minor CVD events, incidence rates marginally decreased from 3.26 to 2.86 events per 100 person-years, respectively. For major CVD events, incidence rates were almost equal (2.54 and 2.76 events per 100 person-years).
2. The majority of patients with intermittent claudication died from cancer and not from vascular complications. Death from vascular causes was more predominant among patients experiencing a major CVD event during follow-up. PAD patients with compared to those without prevalent cardiovascular disease showed a tendency for a higher risk to die which did, however, not reach statistical significance.
3. There was a trend for higher risk for interventions at peripheral vessels among patients who had already undergone an intervention when they were enrolled in the CAVASIC Study than for patients without invasive treatment at baseline.

First, overall mortality, cardio- and cerebrovascular morbidity and mortality in PAD patients in general should be considered. The earlier TASC estimates resulted from population-based studies from the 1990s that have investigated the association between peripheral arterial disease and cardiovascular complications^1^,^17^,^18^. It became evident that an impaired ABI significantly increased the risk for myocardial infarction, angina and stroke. Additionally, overall and CVD mortality was also higher among participants with low ABI values compared to individuals with normal ABI values. The authors pointed out that “all circulatory causes” were causative for the high mortality rates^17^. Similar observations were made by the Whitehall Study which used the Rose questionnaire instead of ABI measurement^19^.

Although the study designs of these studies are not comparable with our study we still recognize that the overall five year mortality (n = 22, 8.6%) in our study population was markedly lower than the predicted overall mortality of 30% within five years in the TASC consensus documents. Considering the fact that TASC recommendations were mostly built on the basis of population-based studies we should have observed at least comparable mortality numbers in our population of IC patients with a relevant atherosclerotic burden. Noteworthy seems the fact that merely 8 out of these 22 patients from our study died due to vascular causes within five years (3.1%). This is much lower than proposed in the TASC consensus document where the vascular mortality is stated to be 23% for five years of follow-up. Even during the entire follow-up time only 13 of our patients (5.1%) died from vascular complications, the vast majority due to heart failure. Cancer was the leading cause of death among our study population. The decreased cardiovascular complication rate might be due to a significant improvement in medical treatment of PAD patients. This is in line with a time-dependent change in plaque composition of atherosclerotic plaques that were harvested during carotid endarterectomy over the last decade that goes hand in hand with improvement in risk factor management^20^.

It is well known that incidence and prevalence rates of peripheral arterial disease and notably of intermittent claudication are strongly dependent on age and sex as mentioned in the TASC recommendations^5^,^6^,^21^,^22^. The proposed five year outcome of patients with intermittent claudication in TASC does not contain detailed information on various patient age strata. Due to small numbers we did not have the opportunity to generate outcome analysis for different age groups in our analysis. Therefore all reported hazard ratios were adjusted for age.

The percentage of non-fatal cardiovascular events of 18% within five years observed in the CAVASIC Study was comparable with the estimated numbers derived by TASC II (20%). Nevertheless, definitions of cardiovascular events changed in the meanwhile by introducing more sensitive diagnostic instruments such as high sensitivity-troponin. Furthermore, indications for interventions liberalized. To rule out a bias in diagnostics and indications we focused on relevant cardio- and cerebrovascular complications in the endpoint assessment. According to this, about a third of all patients suffered a CVD event during the entire follow-up in the CAVASIC Study. We can only speculate why our population had a better survival than expected from the literature. One explanation could be that patients indeed experience cardiovascular complications but they more often survive these events due to improved medical care. The extended life time on the other hand carries the risk of cancer development which then accounts for the majority of deaths.

A variety of studies investigated cancer mortality among PAD patients^23^,^24^,^25^. Fiotti *et al*. examined 223 patients with intermittent claudication and 446 control subjects between 1974 and 1998. The overall mortality was significantly higher among PAD patients compared to control subjects (3.99 vs. 2.53 deaths for 100 patients per year, respectively). Mortality from malignant disease was the most common cause of death among patients with intermittent claudication (1.74 events per 100 patient-years). The incidence of cancer mortality was significantly higher in the PAD group compared to controls (1.74 vs. 0.84 events, per 100 patient-years, p = 0.05)^23^. In a series of 109 patients with intermittent claudication, Taute and colleagues reported that 39% of patients died from vascular events and 36% from cancer. The authors pointed out that 17 out of 20 cancer cases were related to smoking^24^.

The reasons why patients with IC carry a high risk for cancer development are not entirely understood. It might be that with muscle ischemia following the impaired arterial circulation cancer development is supported^25^. Cancer counted for the majority of deaths in our study population. Smoking certainly was a relevant factor for disease development and progression among our patients. Still, we observed a multitude of different types of malignant diseases, which might not solely be caused by smoking at first glance.

Since death due to cancer plays a pivotal role in our patient group, possible bias due to competing risk has been avoided by applying competing risk regression analysis. This has recently been demonstrated to be especially important when two or more major causes of death play a role in the analyzed population and when follow-up time is very long^26^,^27^.

In a next step overall mortality and CVD outcome in PAD patients with and without concomitant cardiovascular disease should be discussed. The Cardiovascular Health Study in 1999 showed that individuals with impaired ABI of less than 0.9 and concomitant cardiovascular disease presented with an overall mortality of 32.3% in a six year follow-up^1^. In 2009, Zeymer and colleagues published results from the REACH registry comparing patients with IC (n = 483) with patients with IC plus concomitant CVD (n = 479)^28^. Interestingly, after 24 months of follow-up, overall and vascular mortality were not significantly different in the two groups (overall mortality 5.5% for patients with IC only vs. 4.6% for IC + CVD, vascular mortality 3.9% vs. 3.7%, respectively). In our study PAD patients with compared to those without prevalent cardiovascular disease showed a tendency for a higher risk to die and suffering from incident cardiovascular events. This, however, did not reach statistical significance which was most probably caused by limited statistical power.

Finally, the local PAD outcome in PAD patients with and without PAD intervention at baseline was investigated. The prognosis of the limb is mainly influenced by the degree of arterial occlusive disease. Only a few studies investigated PAD disease progression. In 1996 Weitz and Byrne stated that about 70% of patients suffering from intermittent claudication stayed stable or even improved in a total follow-up time of five and ten years, respectively^29^. The historical data on disease progression were confirmed in the study at hand. We found that 67% of patients stayed stable or improved during the first five years of follow-up, and 61% during the entire follow-up period (median 7 years), respectively.

The early years of the new millennium were significantly influenced by upcoming endovascular treatment techniques. We therefore made a closer look at type and complexity of interventions. We found that surgical and endovascular treatment techniques were almost equally distributed among our patients. A relevant number of patients received both treatment techniques in different combinations (endovascular treatment prior to surgery, hybrid procedures, endovascular treatment in the course of time after surgery). In a population-based study from the 1970ies to 1980ies, Farkouli and colleagues showed that about 20% of PAD patients undergoing first time surgery for revascularization required an ipsilateral amputation, and 26% required at least one additional ipsilateral revascularization procedure. As expected, patients undergoing aortoiliac or aortofemoral surgery were less likely to require an amputation than patients with a more distal revascularization procedure (16% versus 26%, p = 0.03)^30^. Interestingly, the comparison between patients with and without revascularization procedure at baseline in our study population revealed a tendency towards a higher risk for interventions during follow-up for those with a revascularization procedure at baseline. Patients without intervention at baseline received a relevant number of PAD interventions in the first two years of follow-up and reached a plateau thereafter. In contrast, patients with previous PAD intervention had a more or less constant number of re-interventions every year. At three years, patients without PAD intervention at baseline stayed stable whereas pretreated patients still needed further interventions in the remaining follow-up years.

The CAVASIC Study has strengths and limitations. It offers not only cross-sectional data but delivers prospective long-term follow-up data of a well-defined population of patients with intermittent claudication. One major advantage of the study is the nearly complete follow-up information of the entire study population. In addition, IC diagnostics was very detailed to rule out misdiagnosis for patients’ symptoms. One limiting factor is that our study population comprises only men of Caucasian origin. Moreover, we did not prospectively follow control subjects. The initial study design focused on overall mortality and cardiovascular complications among patients with IC. Therefore we renounced to reevaluate controls.

## Conclusions

TASC I and II proposed that quality of life and life expectancy of patients suffering from intermittent claudication is significantly influenced by cardiovascular complications and vascular mortality. The paper at hand delivers prospective data of a well-defined cohort of patients with IC. This high risk population still suffered from a relevant number of incident minor and major cardiovascular complications. Overall cardiovascular mortality was, however, much lower than expected from literature. The majority of patients died due to cancer. The fact that cancer mortality nowadays exceeds cardiovascular mortality should be taken in consideration in clinical surveillance programs of PAD patients. PAD disease progression and local PAD outcome did not significantly change over the last decades. Numbers of interventions and major amputations are comparable with data from the 1990ies.

## Additional Information

**How to cite this article:** Rantner, B. *et al*. The fate of patients with intermittent claudication in the 21st century revisited – results from the CAVASIC Study. *Sci. Rep.* **7**, 45833; doi: 10.1038/srep45833 (2017).

**Publisher's note:** Springer Nature remains neutral with regard to jurisdictional claims in published maps and institutional affiliations.

## Figures and Tables

**Table 1:** Baseline characteristics of patients with intermittent claudication and available follow-up information, further stratified into those with and without prevalent cardiovascular disease.

	Total group n = 255	PAD + CVD (n = 72)	PAD only (n = 183)	P-value
Age (years)	59 ± 6 [55;60;63]	60.1 ± 5.6 [56.5;61.0;64.5]	58.0 ± 6.7 [53.0;58.0;63.0]	0.02
Body Mass Index (kg/m^2^)	26.8 ± 3.9	28 ± 4.0	26.3 ± 3.8	0.001
Smoking (smokers/former smokers/non-smokers), n (%)	134/106/12 (53;42;5)	31/39/2 (43;54;3)	103/67/10 (57;37;6)	0.05
Diabetes Mellitus, n (%)	39 (15)	16 (22)	23 (13)	0.05
Total cholesterol (mg/dL)	205 ± 41	191 ± 42	211 ± 39	<0.001
LDL cholesterol (mg/dL)	132 ± 37	120 ± 31	137 ± 38	0.001
HDL cholesterol (mg/dL)	49.4 ± 13.6 [41.0;48.0;55.0]	46.6 ± 10.9 [39;46;52]	50.5 ± 14.4 [42;48;57]	0.04
Triglycerides (mg/dL)	173 ± 123 [94;135;213]	183 ± 158 [101;131;231]	169 ± 106 [93;139;209]	0.72
C-reactive protein (mg/L)	6.4 ± 10.9 [2.2;1.4.2;7.0]	5.6 ± 4.6 [2.2;4.5;7.1]	6.8 ± 12.5 [2.2;1.4.1;6.9]	0.71
Creatinine (mg/dL)	0.98 ± 0.2 [0.87;0.96;1.1]	1.01 ± 0.2 [0.89;1.0;1.1]	0.97 ± 0.2 [0.85;0.95;1.01]	0.03
eGFR (mL/min/1.73 m^2^)	85 ± 15	81 ± 14	86 ± 15	0.02
Albumin (g/dL)	4.4 ± 0.5	4.5 ± 0.5	4.4 ± 0.4	0.22
Systolic blood pressure (mmHg)	151 ± 20 [135;150;165]	148 ± 21 [130;150;165]	152 ± 19 [140;150;165]	0.20
Diastolic blood pressure (mmHg)	83 ± 10 [80;80;90]	81 ± 10 [75;80;90]	84 ± 10 [80;80;90]	0.04
Hypertension, n (%)^a^	222 (87)	69 (96)	153 (84)	0.009
Ankle-brachial index^b^	0.72 ± 0.23	0.72 ± 0.23	0.72 ± 0.24	0.84
Intervention due to PAD, n (%)	118 (46)	36 (50)	82 (45)	0.45
ACE inhibitor, n (%)	79 (31)	33 (46)	46 (26)	0.002
Beta blocker, n (%)	47 (18)	27 (38)	20 (11)	<0.001
Calcium antagonist, n (%)	40 (16)	18 (25)	22 (12)	0.01
Angiotensin receptor blockers, n (%)	26 (10)	11 (15)	15 (9)	0.11
Diuretics, n (%)	55 (22)	25 (35)	30 (17)	0.002
Statin use, n (%)	108 (42)	48 (67)	60 (34)	<0.001
Anticoagulants, n (%)	18 (7)	10 (14)	8 (5)	0.009
Antiplatelet medication, n (%)	193 (76)	60 (83)	133 (75)	0.14
Cardiovascular disease (CVD) at baseline, n (%)	72 (28)	72 (100)	n.a	n.a.

Data are presented as mean ± standard deviation (SD) [25^th^, 50^th^ and 75^th^ percentile in case of non-normal distribution] or number (%) (=percent considering missing values). eGFR denotes glomerular filtration rate calculated according to the CKD-EPI equation^16^. ^a^Hypertension was defined as systolic blood pressure ≥ 140 mmHg and/or diastolic blood pressure ≥ 90 mmHg, and/or receiving antihypertensive treatment. ^b^The lowest ABI value from 4 sites was used for data analysis^13^,^14^. Individuals with ABI values > 1.30 were excluded from analysis on ABI. n.a. (not applicable).

**Table 2:** Causes of death of patients with intermittent claudication and available follow-up information, illustrated for the first 5 years and the entire follow-up time.

	First 5 years of follow-up	Entire follow-up time
**Cause of death**		
**Vascular**	**n = 8**	**n = 13**
Heart and/or renal failure	5	10
Myocardial infarction	2	2
Sudden cardiac death	1	1
**Cancer**	**n = 10**	**n = 20**
Oropharynx-, larynx-, bronchial carcinoma	5	9
Gastrointestinal carcinoma (pancreatic-, hepatocellular-, colon carcinoma)	4	5
Prostate carcinoma		2
Carcinoma of unknown origin	1	3
Squamous-cell carcinoma		1
**Infectious disease**	**n = 1**	**n = 3**
Pneumonia	1	2
Sepsis		1
**Unknown reason**	**n = 3**	**n = 5**

**Table 3:** Numbers of cumulative major and minor cardiovascular events in patients with intermittent claudication during the prospective follow-up, shown for the first 5 years and the entire follow-up time.

	First 5 years of follow-up	Entire follow-up time
**Major cardiovascular events**	**N of events = 25**	**N of events = 38**
Fatal cardiovascular events (ICD10 Codes I00-I99)	8^b^	13^b^
Non-fatal myocardial infarction (MI)^a^	14	21
Ischemic cerebral infarction^a^	4	6
**Minor cardiovascular events**	**N of events = 31**	**N of events = 38**
Percutaneous transluminal coronary angioplasty (PTCA)^a^	11	13
Aortocoronary bypass	9	10
Angiographically proven coronary stenosis (CAG) ≥ 50%^a^	4	5
Transient ischemic attack (TIA)^a^	6	9
Carotid endarterectomy (CEA)^a^	1	1

^a^Two patients had a TIA (=first event) and an ischemic stroke (=second event), 1 patient had a CAG (=first event) and an ischemic stroke (=second event); 1 patient had a PTCA (=first event) and an ischemic stroke (=second event); 1 patient had a CEA (=first event) and a MI (=second event) and 1 patient had a TIA (=first event) and a MI (=second event): n = 6. In these patients, the first event was included in the minor and the second event in the major composite cardiovascular endpoint, respectively. ^b^Two patients (one within the first five years) suffered a fatal cardiovascular event and had a non-fatal event before and thus the non-fatal event was considered in the analysis of the major cardiovascular endpoint.
